# Neurodevelopmental outcomes of very low birth weight preterms in preschool childhood: a prospective cohort study

**DOI:** 10.1186/s13052-023-01467-y

**Published:** 2023-05-12

**Authors:** Nadia Battajon, Chiara Bechini, Federica De Osti, Anna Galletti, Anna Chiara Frigo, Paola Lago

**Affiliations:** 1 Neonatal Intensive Care Unit and High-Risk Follow up program, Cà Foncello Regional Hospital, Azienda ULSS 2 Marca Trevigiana Piazzale Ospedale, 1, Treviso, 31100 Italy; 2 Department of Cardiac-Thoracic-Vascular Sciences and Public Health, University of Padua, Padua, Italy

**Keywords:** Neurological development, Neurodevelopmental assessment, Disability, Very low birth weight

## Abstract

**Background:**

Preterm birth is a risk factor for a child’s neurological development. Preterm children have unusual neurodevelopmental profiles with executive, visual-motor functions, fine and gross motor skills, language and behavior that affect learning. In this study, we analyzed the neurodevelopmental outcomes of a cohort of very low birth weight infants admitted to the Treviso Neonatal Intensive Care Unit (NICU) between 2014 and 2016 and followed up to preschool childhood.

**Method:**

This is a prospective cohort study. Infants were followed at birth and after NICU discharge at two- and four-year follow-ups. The two-year assessment was conducted with Bayley III, and at four years with the Wechsler Preschool and Primary Scale of Intelligence - III scales and Movement Assessment Battery for Children − 2.

**Results:**

The cohort consisted of 207 subjects with a mean gestational age of 28.9 weeks, and a mean birth weight of 1097.2 g. At two years of age, children without disabilities were 90 (59.6%), those with minor disabilities 47 (31.1%), and those with major disabilities 14 (9.3%); at four years, 58.4% of children without previous disabilities, presented problems with verbal tests and manual dexterity: aiming, grasping and balance at movement assessment. There was significant alteration in processing speed (p < 0.001). Furthermore, there was a strong correlation between processing speed and manual dexterity (p < 0.001) and between processing speed and aiming and grasping (p = 0.0059).

**Conclusions:**

We found that more than half the children free of disability at two years, at four years had deficit often involving the oculo-motor coordination and processing speed. These motor profile alterations limit the expression of cognitive abilities and the achievement of expected school performance, thus resulting in behavioral disorders, typical of preterm children. Early professional follow-up could improve the expected educational outcomes.

## Background

The clinical history of preterm infants is characterized by extremely heterogeneous neonatal conditions that predispose over time to outcomes with various degrees of complexity [1, 2].

The care given to preterm newborns in the neonatal intensive care unit (NICU) led to an increase in survival at very low gestational ages (GAs) and neonatal weights, making it necessary to plan a multidisciplinary and continuous follow-up until pre-school childhood [3, 4]. Severe neuromotor, sensorineural and cognitive sequelae are evident in the first years of life, affecting 10–20% of very low birth weight infants (VLBWIs), which occur more frequently at lower GAs [4]. Mild sequelae concerning cognitive, communicative-linguistic, attentional, behavioral delays, gross and fine motor skill delays, which compromise executive functions, memory, and learning, appear to be prominent in pre- and school age [5–11].

Even in children without serious perinatal clinical histories (early neonatal sepsis, severe intraventricular hemorrhage, bronchopulmonary dysplasia (BPD), retinopathy of prematurity (ROP), late neonatal sepsis), there is an alteration of the developmental outcomes in the motor history, [12–14] not associated with brain lesions but with early exposure to the adverse extra-uterine NICU environment, early and repetitive sensory and proprioceptive experiences, which can alter connectivity in the preterm brain [15–17]. These minor motor abnormalities are often overshadowed by other more severe physical and intellectual conditions (low IQ, learning disabilities, attention-deficit/hyperactivity disorder, neuropsychologic deficit, behavior problems). The first step in targeted intervention is to identify and characterize them to initiate adequate management [6, 18].

Misidentification of cognitive problems in school-age children with a history of premature birth has often led to the misdiagnosis of learning disabilities and consequently difficulties in providing appropriate support for the child’s abilities.

The objective of this study was to analyze cognitive, motor, attentional and behavioral developmental profiles of VLBWIs at the age of four, in relation to their clinical history and two-year profile, to understand the more common trajectory in the neurodevelopment of VLBWIs.

## Methods

**Fig. 1:**
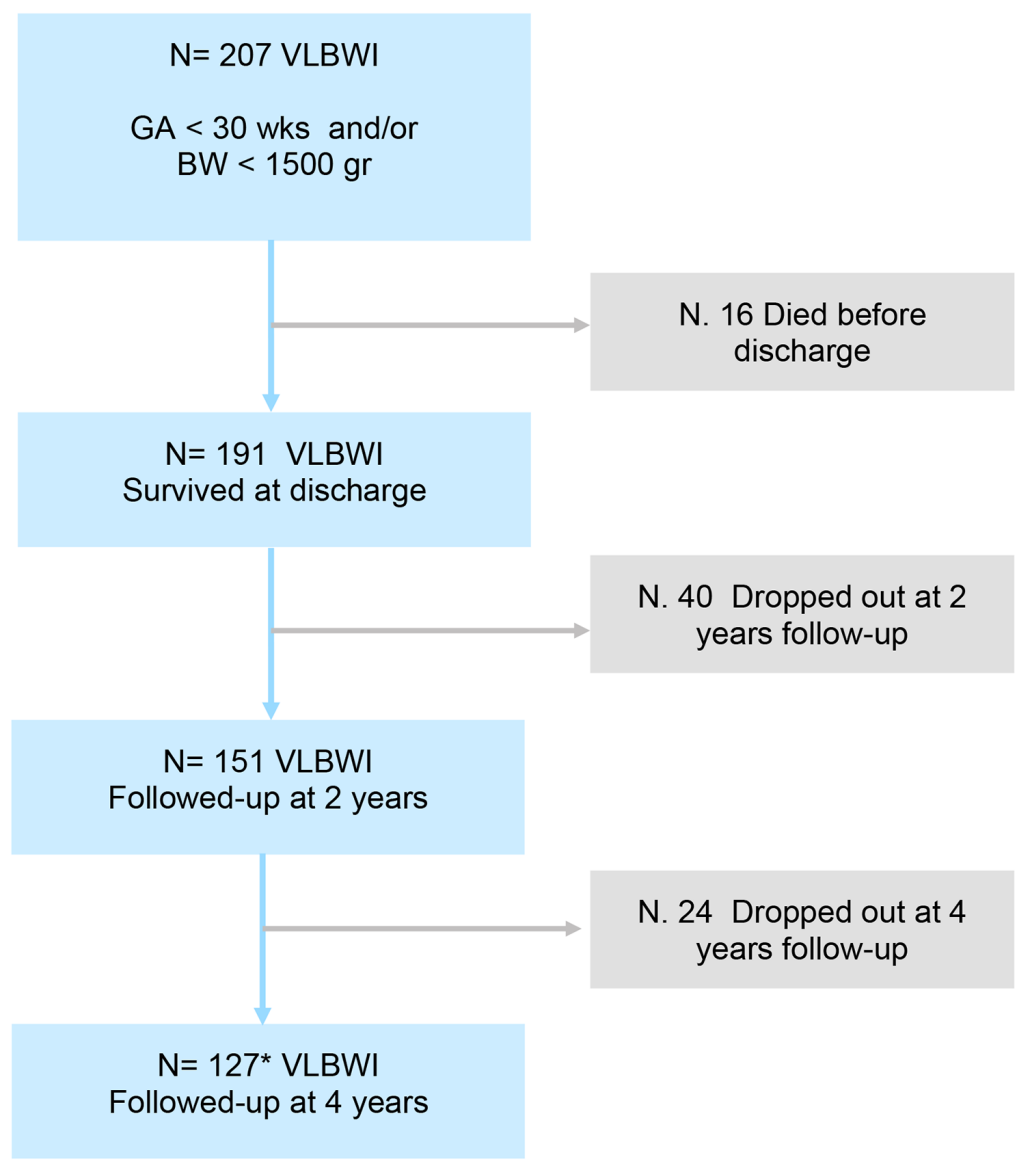
Study Flow chart *3 VLBW not evaluated at 2 years

This is a single tertiary center prospective cohort study. All infants admitted to our NICU in 2014–2016, and January and February 2017 with a GA of less than 30 weeks, or a neonatal weight of less than 1500 g, were included. Those born with malformation syndromes or genetic disease were excluded. This cohort was assessed prospectively during a four-year follow-up (Fig. 1).

At the age of two years, assessment of the development quotient was conducted with the Bayley III scales: cognitive, linguistic and motor administered through direct interaction with the child; socioemotional and adaptive behavior was provided by parents with a self-administered questionnaire. Disability was defined according to the American Academy of Pediatrics as a major disability [5], given by moderate or severe cerebral palsy with Gross Motor Function Classification System (GMFCS) ≥ 2, Bayley III cognitive scores < 70 and GMFCS ≥ 2, vision with a bilateral deficit < 1/10, permanent hearing loss which does not allow the child to communicate, despite a prostheses or cochlear implant. Minor disability was defined as disorders of the motor and postural sphere (clumsiness and motor coordination disorder), with learning disabilities, behavioral disturbances and pathology of adaptive functions.

At the age of four years, the cognitive assessment was conducted with the Wechsler Preschool and Primary Scale of Intelligence - III scales (WPPSI-III), in particular the verbal subtest to evaluate the knowledge of words and the ability to form verbal concepts, and other sub-performance tests that measure the child’s ability to use logical and abstract reasoning and to organize categories, the processing speed to evaluate attention and concentration. Motor assessment was conducted with the Movement Assessment Battery for Children- 2 (mABC 2) scales to detect manual dexterity, aiming, grasping and balance.

In addition, major disability was defined on the WPPSI III and mABC 2 scales with a total IQ score of less than 70 on the first and second scales, along with minor disability and a total IQ score between 70 and 89. Scales have always been administered by neuropsychologists in the Follow-up Service.

All parents gave informed consent, with the study approved by the Clinical Trials Ethics Committee of the Azienda ULSS2 Marca Trevigiana, No. 958 / CE Brand.

### Statistical analysis

Quantitative variables were summarized with mean and standard deviation, and categorical variables with count and percentage of subjects in each category.

The perinatal outcomes potential predictors of disability at 2 and 4 years were evaluated with a univariate cumulative logit model. The outcomes found to be statistically significant at the 5% level, were then considered in a multivariate model with backward selection. The association between gestational age categorized ( 23–25, 26–27 and ≥ 28 wks) and the centile neonatal weight (< 10° / ≥ 10°) with Bayley scale components at 2 years and the WPPSI III and mABC 2 at 4 years, was evaluated with Kruskall-Wallis test in case of quantitative outcome, with chi-square or Fisher’s exact test in case of categorical outcomes. The comparison of the three-gestational age group was followed by pairwise comparison in case of statistical significance at the 5% level.

The correlation between the ability to process information quickly (IVE) of the WPPSI III scale with manual dexterity (DM) and the ability to grasp and aim (ME) using the mABC 2 scale, was evaluated with the Spearman rank correlation test. The level of statistical significance was set at the 5% level.

The statistical analysis was performed with SAS 9.4 (SAS Institute Inc., Cary, NC, USA) for Windows.

## Results

The cohort including 207 VLBWIs was followed until 2021. Characteristics and perinatal outcomes of the population discharged and followed up to two years are reported in Table 1.

**Table 1:** Perinatal characteristics and discharge outcomes of the VLBWI population and followed-up at 2 years

	Study population N. 207	VLBW follow up at 2 years N. 151
	Mean (DS)/n (%)	Mean (DS)/n (%)
Gestational Age (weeks)	28.9 (2.6)	28.9 (2.4)
Birth weight (gr)	1097.2 (281.7)	1101.3 (262.2)
- Percentile	39.3 (27.7)	40.8 (27.0)
- Z score	-0.5 (1.1)	-0.4 (1.0)
Head Circumference (cm)	26.4 (2.3)	26.5 (2.2)
- percentile	45.9 (27.1)	47.8 (26.5)
- Z score	-0.1 (1.0)	-0.1 (1.0)
APGAR score at 5’	8 (1.8)	8.1 (1.6)
Inborn	178 (86.0)	130 (86.1)
Male	107 (51.7)	077 (51.0)
Prenatal steroids	194 (93.7)	146 (96.7)
Cesarean Section	161 (77.8)	114 (75.5)
Respiratory distress	148 (75.1)	118 (78.1)
BPD at 36 weeks	54 (28.1)	036 (23.8)
O2 at home	14 (7.3)	012 (7.9)
PDA	73 (37.8)	60 (39.7)
EOS	13 (6.3)	010 (6.6)
LOS	36 (17.7)	030 (19.9)
NEC	15 (6.7)	11 (7.3)
PVL	10 (5.2)	007 (4.6)
IVH	28 (14.8)	22 (14.6)
IVH ≥ 3	6 (3.5)	3 (2.0)
ROP	42 (27.8)	38 (25.2)
ROP ≥ 2	13 (6.8)	011 (7.3)
Length of stay (days)	61.5 (29.0) *	65.2 (29.0) **
Deaths before discharge or after	16 (7.7)	0 (0)

Patent ductus arteriosus (PDA), Early onset sepsis (EOS), Late onset sepsis (LOS), Necrotizing enterocolitis (NEC), Periventricular leukomalacia (PVL), Intraventricular hemorrhage (IVH), Severe intraventricular hemorrhage (IVH ≥ 3), Retinopathy of prematurity (ROP), Severe retinopathy of prematurity (ROP ≥ 2).

*Excluded patients died

**One missing

Mean and standard deviation (SD) for quantitative variables, number, and percentage of subjects for categorical variables

Patients discharged from the NICU were n = 191 (mortality 7.7%); 20.9% of children did not continue with controls up to 24 months and another 15.9% did not complete the four-year assessment. Drop-outs were mainly due to inability of families to join the high-risk follow-up program, or for transferring to other facilities, going abroad, or for refusal.

Follow-up visits, comprehensive evaluations included neurodevelopmental, pediatric, auxological, nutritional, respiratory, and other special evaluations as needed were tailored to the needs of each individual child and his/her family.

### Results at the two years follow up

The cohort of children evaluated at two years (n = 151) showed no disability in 90 of them (59.6%), a minor disability in 47 (31.1%) and a major disability in 14 children (9.3%) according to the definition described in the Methods. Disability at two years resulted associated in the following neonatal outcomes and perinatal complications: early neonatal sepsis (p = 0.0377), grade ≥ 3 intraventricular hemorrhage (p = 0.0245), BPD (p = 0.0130), ROP (p = 0.0342), late neonatal sepsis (p = 0.0180), and length of hospitalization (p < 0.0001) (Table 2). Using multivariate analysis, only the length of stay was seen as predictive.

**Table 2:** Association of the degree of disability at 2 and 4 years and outcomes at discharge

	Disability at 2 years (n = 151)					Disability at 4 years (n = 127)				
	No (N = 90)	Minor (N = 47)	Major (N = 14)	OR (95% CI)	p- value	No (N = 42)	Minor (N = 60)	Major (N = 25)	OR (95% CI)	p- value
**BPD 36 wks N (%)**										
Presence vs. Absence	16 (17.8)	13 (27.7)	07 (50.0)	2.511 (1.214; 5.194)	**0.0130**	05 (11.9)	16 (26.7)	08 (32.0)	2.258 (1.022; 4.991)	**0.0441**
**PVL N (%)**										
Presence vs. Absence	04 (4.4)	01 (2.1)	02 (14.3)	1.608 (0.379; 6.828)	0.5197	01 (2.4)	05 (8.3)	00 (0.0)	1.000 (0.215; 4.662)	1.0000
**IVH N (%)**										
1–2 vs. 0	09 (10.0)	05 (10 0.6)	05 (35.7)	2.525 (1.006; 6.340)	**0.0245**	04 (9.5)	06 (10.0)	06 (24.0)	2.258 (0.833; 6.119)	0.2683
3–4 vs. 0	0 (0)	2 (4.3)	1 (7.1)	9.641 (1.078; 86.193)		00 (0 0.0)	02 (3.3)	00 (0.0)	1.582 (0.114; 21.958)	
**ROP N (%)**										
1 vs. 0	16 (17.8)	15 (31.9)	06 (42.9)	2.515 (1.221; 5.181)	**0.0342**	09 (21.4)	15 (25.0)	06 (24.0)	1.136 (0.526; 2.454)	0.9319
2 vs. 0	00 (0.0)	01 (2.1)	00 (0.0)	5.038 (0.128; 198.734)		00 (0.0)	01 (1.7)	00 (0.0)	1.470 (0.036; 59.260)	
**EOS N (%)**										
Presence vs. Absence	01 (1.1)	09 (19 0.1)	00 (0.0)	3.622 (1.076; 12.196)	**0.0377**	02 (4.8)	03 (5.0)	02 (8.0)	1.448 (0.346; 6.059)	0.6123
**LOS N (%)**										
Presence vs. Absence	13 (14.4)	11 (23.4)	06 (42 0.9)	2.536 (1.173; 5.481)	**0 0.0180**	06 (14.3)	12 (20.0)	06 (24.0)	1.545 (0.669; 3.571)	0.3083
**Total Length of stay (day) Mean (SD)**										
Per day of increase	57.4 * (21 0.2)	71.1 (30.1)	95.0 (43 0.6)	1.028 (1.016; 1.040)	**< 0.0001**	56.6 (28.5)	66.6 (23.2)	72.4 (29.1)	1.017 (1.005; 1.031)	**0.0077**

*1 Missing

ORs and 95% CI obtained with univariate ordinal logistic regression

At two years, the Bayley motor scale resulted worse in the lowest GA groups (p = 0.0282). No statistically significant difference emerged in the distribution of disability classes at two years between AGA (Adequate for Gestational Age and SGA (Small for Gestational Age) classes, defined as birth weight less than the 10th percentile, according to World Health Organization charts (p = 0.4282) (Table 3).

**Table 3:** Distribution of disability and scores on Bayley III assessments by gestational age group and centile neonatal weight at 2 years of age

	Gestational age class					Centile neonatal weight				
	23–25 (N = 15)	26–27 (N = 40)	≥ 28 (N = 96)	Total (N = 151)	P Value	< 10° (SGA) (N = 28)	≥ 0° (AGA) (N = 123)	Total (N = 151)	P Value	
Missing	00 (00.0%)	00 (00.0%)	00)	00 (00.0%)	0.0576	00 (00.0%)	00 (00.0%)	00 (00.0%)	0.4282	
0 no disability	04 (26.7%)	25 (62.5%)	61 (63.5%)	90 (59.6%)		14 (50.0%)	76 (61.8%)	90 (59.6%)		
1 minor disability	08 (53.3%)	11 (27.5%)	28 (29.2%)	47 (31.1%)		10 (35.7%)	37 (30.1%)	47 (31.1%)		
2 major disability	03 (20.0%)	04 (10.0%)	07 (7.3%)	14 (9.3%)		04 (14.3%)	10 (08.1%)	14 (09.3%)		
**Cog Compos**										
N (N Missing)	15 (0)	35 (5)	86 (10)	136 (15)		27 (1)	109 (14)	136 (15)		
Mean (SD)	98.0 (11.9)	98.1 (9.6)	101.5 (11.4)	100.3 (11.1)	0.1035	98.3 (13.5)	100.8 (10.4)	100.3 (11.1)	0.4138	
**Lang compos**										
N (N Missing)	13 (2)	29 (11)	79 (17)	121 (30)		21 (7)	100 (23)	121 (30)		
Mean (SD)	95.1 (10.8)	92.6 (10.5)	95.6 (11.1)	94.8 (10.9)	0.5028	91.7 (11.9)	95.5 (10.7)	94.8 (10.9)	0.1701	
**Motor compos**										
N (N Missing)	14 (1)	31 (9)	82 (14)	127 (24)		24 (4)	103 (20)	127 (24)		
Mean (SD)	89.5 (7.6)	96.1 (12.1)	97.2 (9.4)	96.1 (10.2)	**0.0282**	93.2 (8.4)	96.8 (10.5)	96.1 (10.2)	0.1038	
**Social compos**										
N (N Missing)	10 (5)	19 (21)	75 (21)	104 (47)		17 (11)	87 (36)	104 (47)		
Mean (SD)	96.5 (10.0)	106.3 (18.5)	103.8 (25.0)	103.6 (22.9)	0.3789	103.2 (24.0)	103.6 (22.8)	103.6 (22.9)	0.7543	
**GAC compos**										
N (N Missing)	10 (5)	20 (20)	75 (21)	105 (46)		17 (11)	88 (35)	105 (46)		
Mean (SD)	93.1 (18.8)	100.0 (10.3)	97.0 (24.7)	97.2 (22.1)	0.3871	94.3 (28.3)	97.8 (20.9)	97.2 (22.1)	0.0689	

### Results at the four years follow up

Assessment at four-years, 127 out of 151 children followed until two years were evaluated, showing major disability in 25 (19.7%), a minor disability in 60 (47.2%), or no disability in 42 (33.1%). Statistical analysis showed that the disability was only associated with BPD (p = 0.0441) and length of hospitalization (p = 0.0077) (Table 2). Using multivariate analysis, only the length of stay was seen as predictive. Even at age four, considering AGA and SGA groups, there was no difference in the incidence of disabilities (p = 0.2689) (Table 4).

**Table 4:** Distribution of disability and scores on WPPSI and mABC 2 assessments by year gestational age group and centile neonatal weight at 4 years of age

	Gestational Age					Centile neonatal weight			
	23–25 (N = 13)	26–27 (N = 35)	≥ 28 (N = 79)	Total (N = 127)	P Value	< 10° (SGA) (N = 26)	≥ 10° (AGA) (N = 101)	Total (N = 127)	P Value
Missing	00 (00.0%)	00 (00.0%)	01 (01.3%)	01 (00.8%)		01 (03.8%)	00 (00.0%)	01 (00.8%)	
0 no disability	05 (38.5%)	08 (22.9%)	28 (35.9%)	41 (32.5%)		07 (28.0%)	34 (33.7%)	41 (32.5%)	
1 minor disability	05 (38.5%)	17 (48.6%)	38 (48.7%)	60 (47.6%)		10 (40.0%)	50 (49.5%)	60 (47.6%)	
2 major disability	03 (23.1%)	10 (28.6%)	12 (15.4%)	25 (19.8%)	0.3775	08 (32.0%)	17 (16.8%)	25 (19.8%)	0.2344
**ICV - WPPSI**									
N (N Missing)	10 (3)	28 (7)	64 (15)	102 (25)		20 (6)	82 (19)	102 (25)	
Mean (SD)	113.6 (8.0)	109.2 (9.9)	115.1 (10.9)	113.3 (10.6)	**0.0170**	113.9 (12.9)	113.2 (10.1)	113.3 (10.6)	0.4777
**IVP- WPPSI**									
N (N Missing)	10 (3)	28 (7)	67 (12)	105 (22)		20 (6)	85 (16)	105 (22)	
Mean (SD)	107.8 (8.6)	107.6 (16.3)	112.0 (12.8)	110.4 (13.6)	0.1992	110.7 (14.5)	110.4 (13.4)	110.4 (13.6)	0.8959
**IVE- WPPSI**									
N (N Missing)	10 (3)	28 (7)	67 (12)	105 (22)		20 (6)	85 (16)	105 (22)	
Mean (SD)	81.8 (18.9)	81.0 (15.4)	83.1 (19.3)	82.4 (18.1)	0.8216	83.1 (19.8)	82.2 (17.8)	82.4 (18.1)	0.9381
**QI TOT- WPPSI**									
N (N Missing)	10 (3)	28 (7)	64 (15)	102 (25)		20 (6)	82 (19)	102 (25)	
Mean (SD)	109.1 (8.5)	107.2 (13.7)	113.5 (13.0)	111.3 (13.0)	**0.0508**	112.4 (14.8)	111.1 (12.6)	111.3 (13.0)	0.6276
**DM - mABC**									
N (N Missing)	10 (3)	28 (7)	67 (12)	105 (22)		20 (6)	85 (16)	105 (22)	
Mean (SD)	9.9 (4.5)	8.6 (2.8)	11.9 (4.1)	10.8 (4.1)	**0.0013**	11.5 (4.0)	10.7 (4.1)	10.8 (4.1)	0.4270
**ME- mABC**									
N (N Missing)	10 (3)	26 (9)	66 (13)	102 (25)		20 (6)	82 (19)	102 (25)	
Mean (SD)	6.4 (2.4)	7.8 (3.7)	9.1 (3.7)	8.5 (3.7)	**0.0291**	8.3 (3.5)	8.5 (3.8)	8.5 (3.7)	0.8783
**E- mABC**									
N (N Missing)	10 (3)	27 (8)	67 (12)	104 (23)		20 (6)	84 (17)	104 (23)	
Mean (SD)	13.8 (5.3)	15.5 (4.7)	16.6 (4.0)	16.1 (4.3)	**0.0499**	16.4 (4.5)	16.0 (4.3)	16.1 (4.3)	0.5170
**TOT- mABC**									
N (N Missing)	10 (3)	26 (9)	66 (13)	102 (25)		20 (6)	82 (19)	102 (25)	
Mean (SD)	10.6 (4.3)	11.5 (4.1)	14.4 (4.5)	13.3 (4.6)	**0.0031**	13.8 (4.8)	13.2 (4.6)	13.3 (4.6)	0.5812

Definitions: ICV = verbal tests, IVP = ability of logical and abstract reasoning and the ability to organize categories, IVE = processing speed, IQ = intellectual quotient, DM = manual dexterity, ME = aiming and grasping, E = balance

Considering the results of the cognitive (WPPSI-QI TOT) and motor assessments (mABC 2 TOT) in relation to GA groups, progressively worse performance was noted in relation to reduction of the GA (Table 4).

**Fig. 2:**
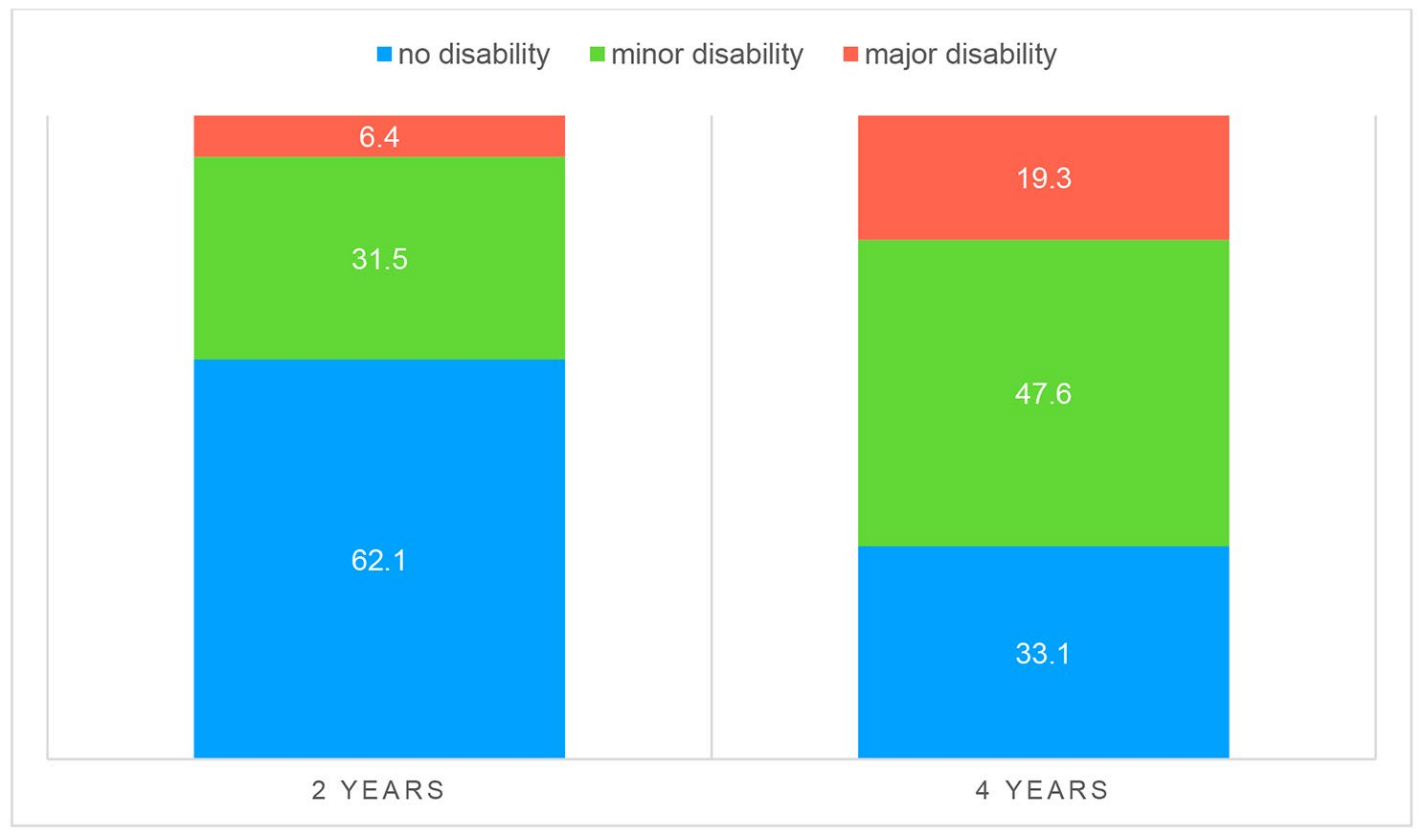
Disability distribution at 2 and 4 years (n = 124 with both evaluations)

The analysis of the conjoint distribution of disability at age of two and four years revealed how children without disabilities at the age of two (n = 77, 62.1%) developed impairments at the age of four in 58.4% of cases (p < 0.0001) (Fig. 2).

**Fig. 3:**
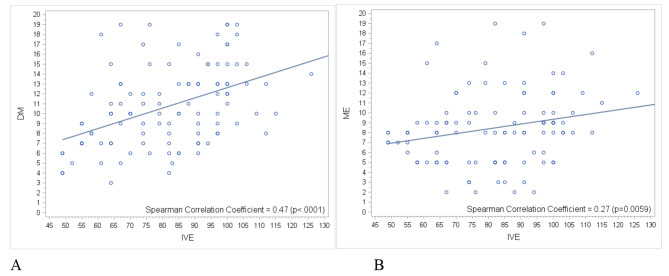
Correlation between processing speed (IVE) and manual dexterity (DM) (A) processing speed (IVE) and aiming and grasping (ME) (B)

Statistical analysis revealed significant correlation between processing speed and manual dexterity with Spearman’s coefficient = 0.47 (p < 0.0001) and between processing speed and aiming and grasping with Spearman’s coefficient = 0.27 (p < 0.0001). Modest processing speed scores also correlated with modest scores in manual dexterity and the ability to aim and grasp (Fig. 3).

## Discussion

The results of our study show that the neurodevelopmental assessment at the age of two is not indicative of the neurodevelopmental profile at pre-school age. This is mostly due to poor processing speed which impacts the total cognitive score with WPPSI scales.

We confirm how perinatal clinical history (neonatal sepsis, grade ≥ 3 IVH, BPD, ROP and length of hospitalization) significantly influence the degree of disability at two years, while at four years the neurodevelopmental outcome is compromised by BPD and the length of hospitalization.

According to our results, Do et al. confirmed that perinatal risk factors became less impacting on disabilities in the long-term, as the environment acquires an even greater influence [19]. In contrast, the study based on the EPIPAGE-2 cohort showed that at five years, only GA correlated with the neurodevelopment outcome [20].

Length of hospital stay and gestational age are strictly related and define the complexity of the perinatal phase. Furthermore, our analysis excluded a correlation between SGA children and neurodevelopmental outcomes, as already extensively described [20].

We demonstrated a clear shift in the incidence of disabilities at the four-year evaluations: about half of children completely free from disability at two years of age, showed a disability related to fine motor skills that impacted an alteration in processing speed at four years. This evidence is confirmed by numerous studies in the literature, however without clarifying the reasons for the increase in disability at preschool age. One study conducted in Taiwan on ~ 6,000 children, born between 2002 and 2009, found that one-fifth of VLBW preterm children with abnormal neurodevelopmental outcomes at 5 years had normal or borderline neurologic and developmental assessments at 2 years. [21].

In a recent Swedish study as well, 22% of preterm infants, examined at 2.5 years without problems, had cognitive impairments first detected at 6.5 years [22].

We should stress that the use of the Bayley III scales administered to two-year children may not be predictive of the preschool outcome, as it describes the development reached at that time and cannot account for environmental factors or the social and cultural level of the parents, which significantly determines early childhood development [23–26].

Based on stratified results by groups of GA, at two years the motor deficit of the composite type (fine and gross) is related to lower GA and it is confirmed at four years in all the sub scales of mABC 2. This was also reported by a Swedish study, conducted in a cohort of 400 children born at fewer than 27 GA evaluated at 6.5 years: motor coordination disorder was present in 37% and borderline motor function was present in 15%. In these children are more likely to have behavioral and intellectual comorbidity. It is necessary to identify motor disorders correctly as early as possible and reduce the negative impact that they may have for future learning [14]. Moreover, much of the literature over the past ten years has found that fine motor disorders closely affect the quality of life in premature children [27, 28]; for this reason, the need to extend follow-up to at least preschool age is reiterated [11, 24].

In our study, an impaired neurodevelopmental profile was observed as early as age four in children who showed no disability at age two, specifically due to low processing speed leading to a lower total cognitive score on the WPPSI. In fact the processing speed is linked to handling information quickly, along with implications for attention, memory, and academic results. A close correlation was sought between processing speed wih the WPPSI scale and scores with mABC 2 assessments, confirming that low processing speed scores correlate with low scores in manual dexterity, as well as aiming and grasping. This suggests that attentional capacity may not be the primary cognitive problem, but a motor impairment and a difficulty with oculo-motor coordination in the assessment. Children with oculo-motor impairment have less cognitive results and this does not reflect their true abilities. Therefore, for proper assessment of school learning problems, it is necessary to conduct a careful follow-up on all cognitive, motor and behavioral aspects as early as possible to detect the real problem. This allows intervention with appropriate neuropsychological techniques and thus improves school performance.

### Strengths and limitations of our study

One limit of the present study is the high number of dropouts due to poor parental adherence or transferring to another venue before the conclusion of the assessment. The strength is a standardized follow-up program for national and international recommendations and stable professional roles, reducing individual variability in the evaluation itself.

## Conclusions

Alterations in the fine motor profile (oculo-motor coordination, hand coordination, grasp and fine movement) may limit the expression of cognitive abilities and the achievement of expected academic results and cause behavioral disorders, typical of premature births. This study reiterates the need for careful and prolonged follow-up at least until pre-school age, to identify developmental abnormalities correctly, so that proper treatment can be started as early as possible.

## Data Availability

The datasets used or analyzed during the current study are available from the corresponding author on reasonable request.

## Abbreviations

AGA: Adequate for Gestational Age
BPD: Bronchopulmonary Dysplasia
CESC: Clinical Trials Ethics Committee
GA: Gestational Age
GMFCS: Gross Motor Function Classification System
DCD: Motor Coordination Disorder
E: Balance
EOS: Early Onset Sepsis
ICV: Verbal Compréhension Index
IQ: Intelligence Quotient
IVE: Processing Speed Index
IVH: Intraventricular Hemorrhage
IVP: Performance Speed index
LOS: Late Onset Sepsis
mABC: Movement Assessment Battery for Children
ME: Aiming and Grasping
MD: Manual Dexterity
NEC: Necrotizing Enterocolitis
NICU: Neonatal Intensive Care Unit
PDA: Patent Ductus Arteriosus
PVL: Periventricular leukomalacia
ROP: Retinopathy of prematurity
SD: Standard Deviation
SGA: Small for Gestational Age
VLBWIs: Very Low Birth Weight Infants
WPPSI: Wechsler Preschool Primary Scale Intelligence
